# Effect of multidisciplinary interventions in perioperative management center on duration of preoperative fasting: A single-center before-and-after study

**DOI:** 10.20407/fmj.2021-021

**Published:** 2022-01-25

**Authors:** Satoshi Komatsu, Chizuru Yamashita, Tomoaki Yatabe, Naohide Kuriyama, Tomoyuki Nakamura, Osamu Nishida

**Affiliations:** 1 Department of Anesthesiology and Critical Care Medicine, Fujita Health University, School of Medicine, Toyoake, Aichi, Japan; 2 Department of Anesthesiology, Nishichita General Hospital, Tokai, Aichi, Japan

**Keywords:** Enhanced Recovery After Surgery, Large hospital, Perioperative management center, Perioperative complication, Duration of preoperative fasting

## Abstract

**Objectives:**

Our aims were to clarify the actual situation regarding preoperative fasting and determine whether multidisciplinary interventions in a perioperative management center shorten the duration of preoperative fasting.

**Methods:**

The cohort of this before-and-after study comprised patients undergoing elective surgery aged 18 years or older who underwent general anesthesia at one of three stages: after starting a short preoperative fasting protocol (Group A), after the anesthesiologist started explaining the protocol (Group B), and after the start of the perioperative management center (Group C). Instructions on drinking clear fluids were given up to 2 h and 4 h before the start of elective surgery to the first patient on the list (on-time) and to the second and subsequent patients (on-call), respectively. Data were collected retrospectively in Groups A and B and prospectively in Group C.

**Results:**

The study cohort comprised 89 patients in Group A (50 on-time, 39 on-call), 108 in Group B (65 on-time, 43 on-call), and 284 in Group C (182 on-time, 102 on-call). The difference between the instructed and last drinking time was significantly shorter in Group C than Group A (30 [10, 140] vs. 30 [10, 60] vs. 20 [0, 50] min, p=0.003). The duration of fasting was significantly shorter in Group C than Group B (243 [150, 395] vs. 213 [151, 323] vs. 180 [146, 280] min, p=0.01).

**Conclusions:**

Multidisciplinary interventions at the perioperative management center tended to reduce the duration of fasting, suggesting that this approach may contribute to improved compliance.

## Introduction

**Figure 1:**
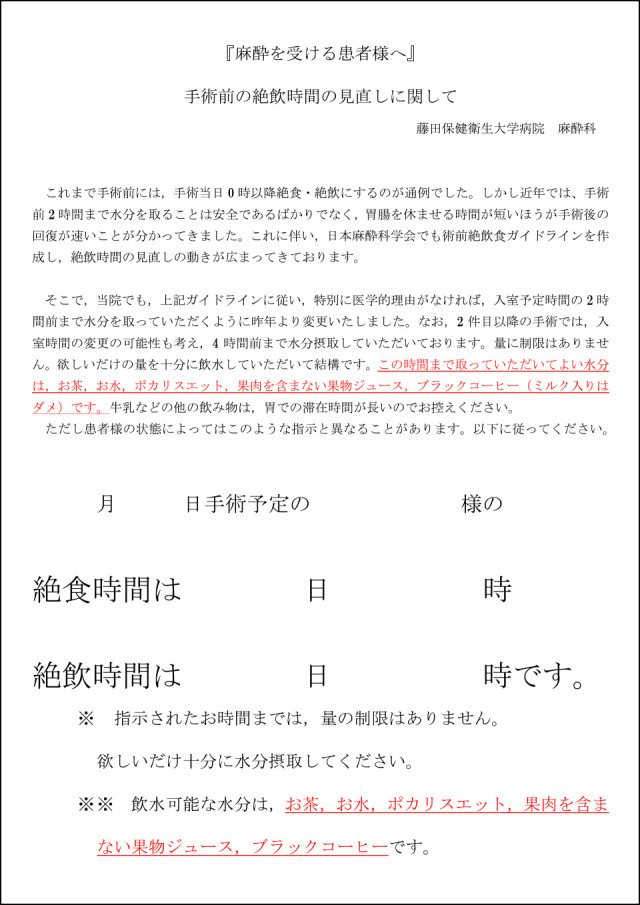
Instructions to the patient. The actual duration of fasting can be entered (in Japanese). We used this document to explain that safety and rapid recovery are best achieved by not limiting the amount or specifying the type of clear fluids drunk preoperatively.

The concept of enhanced recovery after surgery (ERAS) has recently strongly influenced approaches to perioperative management.^1^ ERAS, a European protocol designed to enhance recovery after surgery while maintaining safety, aims to achieve rapid recovery, even after highly invasive surgery. This protocol is designed to reduce reactions requiring invasive interventions, prevent complications, and accelerate postoperative recovery.^1–3^ The concept of ERAS started in the field of gastroenterology, where it was widely practiced, and gradually spread to other fields.^1,4–6^ Some of the 17 items in this protocol are to do with preoperative drinking and carbohydrate intake.^7^ It has been reported that shortening the duration of preoperative fasting can increase patient satisfaction and reduce anxiety on recommencing eating.^8^ It has also been reported that prolonged restriction of drinking contributes to circulatory variations during induction of anesthesia.^9^ Additionally, the Japanese Society of Anesthesiologists’ guidelines for preoperative fasting state that drinking clear fluids up to 2 h before induction of anesthesia is safe.^10^ Despite these guidelines and protocols, prolonged fasting is still customary in many institutions. For example, 50% of anesthesiologists in Europe, Canada, and Australia require abstinence from drinking clear fluids after midnight,^11^ and only 45% of anesthesiologists in Lebanon allow drinking of clear fluids up to 2 h before surgery.^12^ It has also been reported that the fasting time instructed by nurses can be as much as 3 h longer than that instructed by anesthesiologists, and that the actual duration of fasting is even longer than instructed,^13^ making it difficult to promote shortening the duration of preoperative fasting.^14,15^ In our hospital, we started using a protocol incorporating shortening the duration of preoperative restriction of clear fluids in July 2013. The anesthesiologist in charge of anesthesia began to distribute a copy of this protocol and explain it during the preoperative consultation (Figure 1). Additionally, we opened a perioperative management center in September 2015 and initiated multidisciplinary interventions. In this before-and-after study, we aimed to clarify the actual situation regarding restriction of clear fluids in our hospital and to determine whether the duration of preoperative restriction clear fluids was shortened by multidisciplinary interventions.

## Methods

This study was approved by the Ethics Committee of Fujita Medical School (HM20-030). Because this was a retrospective, observational study, the requirement for obtaining written consent was waived. The participants were elective surgical patients aged 18 years or older who underwent general anesthesia during the following three periods: 2 months after the protocol for short preoperative fasting became operational (Group A: 11–30 September 2013 [3 weeks]), 6 months after the anesthesiologist began distributing the instruction manual during preoperative assessment (Group B: 24 March–18 April 2014 [3 weeks]), and 5 years after opening the perioperative management center (Group C: 22 June–11 July 2020 [3 weeks]) (Table 1). The following patients were excluded: patients undergoing electroconvulsive therapy, those with gastrointestinal stenosis (pyloric stenosis, ileus), those with gastrointestinal dysfunction, highly obese patients with a body mass index of 35 or higher in whom securing the airway could be difficult, high-risk pregnant women (in labor, with abnormal fetal heart rate), patients undergoing surgery in the prone position, patients with impaired oral intake due to dementia or decreased level of consciousness, those with missing data, and those who were judged by the anesthesiologist or attending physician to require prolonged fasting. In all groups, the duration of fasting with respect to solid food was until 24:00 on the day before surgery. No specific types of preoperative clear fluid (e.g., water, carbohydrate-containing beverage) were recommended. In general, intake of clear fluids was permitted until 2 h before the scheduled operation time in the first patient on the list (on-time), and until 4 h before the scheduled operation time in the second and subsequent patients (on-call). However, final decisions were left to individual anesthesiologists. The following data were extracted retrospectively from electronic medical records and anesthesia records in Groups A and B, and prospectively in Group C: age, sex, height, weight, department, American Society of Anesthesiologists (ASA) physical status, difference between scheduled and actual time of entering the operation room, difference between instructed and actual last drinking time, duration of fasting, consumption of 200 mL or more of clear fluids, and adverse reactions (vomiting and hypotension). In Group C, the date of the perioperative management center visit, the interval between that visit and the day of surgery, and the presence or absence of a preoperative visit were recorded. The primary endpoint was the duration of fasting, which was defined as the difference between the time of the last drink and that of entering the operation room. The secondary endpoints were the final volume of clear fluids consumed, the difference between the instructed and actual final time of consumption of clear fluids, and decrease in blood pressure during induction. Decreased blood pressure during induction was defined as administration of ephedrine (8 mg or more), phenylephrine (0.2 mg or more), or initiation of continuous catecholamine administration between the start of anesthesia and the start of surgery. Data are presented as median (interquartile range). Statistical analyses were performed using Fisher’s exact test for nominal variables and the Bonferroni method for multiple comparisons. All statistical analyses were performed with EZR (Saitama Medical Center, Jichi Medical University, Saitama, Japan),^16^ which is a graphical user interface for R (The R Foundation for Statistical Computing, Vienna, Austria).

## Results

During the study period, 261 patients in Group A, 401 in Group B, and 424 in Group C underwent general anesthesia. After exclusion of 172 patients in Group A, 293 in Group B, and 140 in Group C, 89 patients in Group A (50 on-time and 39 on-call), 108 in Group B (65 on-time and 43 on-call), and 284 in Group C (182 on-time and 102 on-call) were finally included in the analysis.

### Overall

Table 2 shows the characteristics of all study patients, including on-time and on-call. There were significant differences in sex, height, and weight between Groups A, B, and C (56% vs. 60% vs. 47%, p=0.03; 161 [153, 166] vs. 158 [152, 165] vs. 160 [155, 168] cm, p=0.008; 54 [50, 63] vs. 56 [48, 64] vs. 59 [51, 68] kg, p=0.02, respectively). There were no significant differences in age, ASA status, percentage of general surgery, or percentage of on-call patients. The difference between the instructed and actual last drinking time was significant and was significantly shorter in Group C than in Group A (30 [10, 140] vs. 30 [10, 60] vs. 20 [0, 50] min, p=0.003). The duration of fasting was significantly shorter in Group C than in Groups A and B (243 [150, 395] vs. 213 [151, 323] vs. 180 [146, 280] min, p=0.01). A significantly greater percentage of patients consumed 200 mL or more of clear fluids in Group B than in Group C (9% vs. 19% vs. 2%, p<0.001). There was no vomiting in any of the three groups. The incidence of hypotension was significantly higher in Group C than in Group B (3% vs. 1% vs. 11%, p<0.001).

### Subgroup analysis

#### On-time

There were no significant differences in sex, height, weight, ASA status, or percentage of general surgery between Groups A, B, and C. The findings for the on-time patients are presented in Table 3. The difference between the instructed and last drinking time was significantly shorter in Group C than in Groups A and B (30 [10, 420] vs. 30 [15, 60] vs. 25 [5, 40] min, p=0.003). The differences between the groups in duration of fasting and consumption of ≥200 mL of clear fluid were not significant. The incidence of hypotension was significantly higher in Group C than in Group B (6% vs. 2% vs. 12%, p=0.03).

#### On-call

The findings for the on-call patients are listed in Table 4. Patients in Group B were significantly younger than those in Groups A and C (66 [52, 72] vs. 53 [39, 63] vs. 63 [52, 74] years, p=0.002). There were no significant differences between the groups in sex, height, weight, ASA status, or percentage of general surgery. The difference between the scheduled and actual time of entering the operation room was significantly shorter in Group C than in Groups A and B (6 [–30, 36] vs. 15 [2, 54] vs. –2 [–51, 16] min, p<0.001). Differences between the instructed and last drinking time and duration of fasting were not significant. A significantly higher percentage of patients in Group B than in Groups A and C had drunk ≥200 mL of clear fluids (13% vs. 37% vs. 3%, p<0.001). The incidence of hypotension was significantly higher in Group C than in Group B (0% vs. 0% vs. 9%, p=0.02).

Table 5 shows the findings for consumption of clear fluids in Group C. The interval in days between the perioperative management center visit and surgery or preoperative examination did not differ significantly between the ≤50 mL, 50–200 mL, and ≥200 mL groups.

## Discussion

We conducted a before-and-after study to clarify the actual situation regarding preoperative restriction of clear fluids in our hospital and to test the hypothesis that the duration of preoperative clear fluid restriction can safely be shortened by multidisciplinary interventions. To achieve this, anesthesiologists provided instructions that incorporated a recommended 2 h of fasting period before on-time surgery and 4 h before on-call surgery. We found that patients’ compliance with the duration of fasting tended to improve with multidisciplinary interventions at the perioperative management center. Previous studies have not identified this.^11–15^

Preoperative fasting at the time of anesthesia induction is considered necessary because of the risk of aspiration. However, in the 1980s it was reported that emptying of liquid from the stomach takes approximately 1 h.^17,18^ A meta-analysis concluded that short periods of fasting did not increase the risk of complications over that associated with prolonged fasting.^7^ Prolonged fasting reportedly results in difficulty and discomfort with re-introduction of food.^8^ Currently, guidelines in many countries permit preoperative drinking for up to 2 h before surgery.^19^

There have been studies on the duration of restriction of clear fluids in many countries worldwide. In Lebanon, approximately 90% of anesthesiologists are aware of the ASA recommendations on fasting and are state that they are willing to comply with them. However, only 45% allow fluid consumption up to 2 h before the procedure.^12^ In a study in the Netherlands, 67.8% of patients were asked to fast for a long time, 20.8% being asked to fast for more than 12 h.^20^ In a multinational survey of anesthesiologists in Canada, Australia, New Zealand, and Europe, 85% of anesthesiologists claimed to follow the guidelines; however, approximately 50% did not.^11^ Thus, although some anesthesiologists reportedly instruct fasting for longer than recommended, despite their countries’ guidelines, in this study, fasting instructions were given as per protocol. The difference between the instructed and last drinking time was small (within 40 min) for both on-time and on-call surgery. It was significantly shorter in Group C for on-time surgery and tended to be shorter for on-call surgery.

However, subgroup analysis showed that the duration of fasting was 274 min, even in Group C, particularly for on-call. This was because the instructed drinking time was up to 4 h before the on-call time. The commonest reason for not adhering to the guideline of allowing drinking of water up to 2 h prior to surgery in previous international surveys was concerns regarding variable operation room schedules.^11,12^ However, in the former of these surveys, only about 15% of respondents reported that surgery was often performed earlier than scheduled, and only about 5% reported that this was often a problem. Approximately 15% of anesthetists reported operations being moved to an earlier time than planned. Only 5% of anesthesiologists stated that these schedule changes frequently caused problems.^11^ In the present study, there was very little difference between the scheduled and actual time for entering the operation room for on-call surgery. This finding suggests that anesthesiologists play a role in shortening the duration of abstinence from clear fluids for on-call surgery. However, in the present study, the difference between the instructed and actual last drinking time was significantly shorter in Group C for on-time surgery and tended to be shorter for on-call surgery. These findings suggest that educating patients about drinking clear fluids through multidisciplinary interventions in the perioperative management center setting may be useful in shortening the duration of fasting.

In contrast, there is no clear recommendation regarding the volume of fluid that should be consumed preoperatively. Studies that have been used as the basis for guideline recommendations have varied from citing 100 mL to no limit.^21^ Preoperative drinking of 400 mL of carbohydrate-containing beverages can reportedly reduce insulin resistance.^22^ In accordance with published reports,^23–25^ we recommend that patients drink a full glass (200 mL) of clear fluid. Therefore, in this study, we used 200 mL as the cutoff when examining the volume of clear fluids consumed. Consequently, the percentage of patients who drank ≥200 mL of clear fluid was the highest in Group B (19%) and significantly lower in Group C (2%).

Hypotension during anesthesia is harmful and one-third of hypotensive episodes occur between induction of anesthesia and the initial incision.^26^ Prolonged preoperative fasting has been reported to significantly increase circulatory variability during induction compared with drinking clear fluid up to 2 h before surgery, suggesting that preoperative drinking may reduce hypotension during induction.^9^ In contrast, some researchers have reported that duration of fasting is not associated with hypotension during induction.^27^ Thus, the relationship between preoperative drinking and hypotension during induction is unclear. We hypothesized that drinking large volumes of clear fluids and a short fasting time would minimize hypotension during induction. In the present study, the duration of fasting was 30–60 min shorter in Group C than in Groups A and B, but 7% and 17% fewer patients in Group C than in Groups A and B, respectively, drank more than 200 mL of water. The percentage of patients with hypotension was highest in Group C and lowest in Group B. These results suggest that the volume of water consumed may contribute more strongly than the duration of abstinence from fluids to lowering blood pressure at the time of induction. They also suggest that explaining the policy on consumption of fluids in the ward after admission, as in Group B, might increase the volume of clear fluids consumed. In the future, combining post-hospitalization explanations by ward nurses with multidisciplinary interventions in preoperative management centers may contribute to shortening the duration of fasting and increasing the volume of clear fluids consumed.

This study had some limitations. It was conducted over only 3 weeks, and Groups A and B were studied retrospectively, whereas Group C was studied prospectively. The frequency of hypotension and vomiting and volume of clear fluids consumed may have been influenced by the study design.

## Conclusion

We evaluated the status of preoperative drinking of clear fluids in our hospital. Anesthesiologists provided instructions that incorporated the recommended duration of fasting and patient compliance with that duration tended to improve with multidisciplinary interventions in our perioperative management center. However, this intervention did not affect the percentage of patients who drank more than 200 mL of clear fluids.

## Figures and Tables

**Table1:** Observation period and intervention method for each group

	Group A	Group B	Group C
observation period	11–30 September 2013	24 March–18 April 2014	22 June–11 July 2020
Educator to patients	none	Anesthesiologist	multidisciplinary intervention
Date of education	none	Mostly the day before surgery	Mostly 2 weeks before surgery
Date of fasting instruction	the day before surgery	the day before surgery	perioperative management center
Description	oral	Document and oral	Document and oral

**Table2:** The results for all cases

	Group A (N=89)	Group B (N=108)	Group C (N=284)	P value
Age years	65 [54, 72]	63 [47, 71]	66 [51, 73]	0.28
Sex Female, N (%)	50 (56)	65 (60)	133 (47)	**0.03**
Height cm	161 [153, 166]	158 [152, 165]*	160 [155, 168]*	0.008
Weight kg	54 [50, 63]	56 [48, 64]	59 [51, 68]	**0.02**
Body Mass Index kg/m^2^	21.9 [20.1, 23.9]	22.0 [20.2, 24.3]	22.4 [20.2, 25.7]	0.61
ASA	2 [1, 2]	2 [1, 2]	2 [1, 2]	0.50
General surgery, N (%)	34 (38)	39 (36)	96 (34)	0.74
On-call, N (%)	39 (44)	43 (40)	101 (36)	0.37
The difference between the instructed and last drinking time, min	30 [10, 140]*	30 [10, 60]	20 [0, 50]*	**0.003**
The duration of fasting, min	243 [150, 395]	213 [151, 323]*	180 [146, 280]*	**0.01**
Clear fluid consumption of 200 mL or more, N (%)	8 (9)	20 (19)*	8 (2)*	**<0.001**
Vomit, N (%)	0 (0)	0 (0)	0 (0)	—
Hypotension, N (%)	3 (3)	1 (1)*	30 (11)*	**<0.001**

Bold font: p<0.05, with a significant difference between *.

**Table3:** The results for on-time

	Group A (N=50)	Group B (N=65)	Group C (N=182)	P value
Age years	65 [58, 72]	67 [53, 73]	66 [51, 73]	0.84
Sex Female, N (%)	27 (54)	37 (58)	84 (46)	0.27
Height cm	162 [153, 167]	158 [152, 165]*	161 [155, 169]*	0.122
Weight kg	55 [50, 64]	57 [48, 65]	60 [51, 68]	0.12
Body Mass Index kg/m^2^	22.0 [19.5, 23.9]	22.6 [19.8, 24.3]	22.2 [20.2, 25.6]	0.40
ASA	2 [2, 2]	2 [2, 2]	2 [2, 2]	0.99
General surgery, N (%)	23 (46)	27 (42)	67 (37)	0.5
The difference between the instructed and last drinking time, min	30 [10, 420]*	30 [15, 60]^#^	25 [5, 40]*^#^	**0.003**
The duration of fasting, min	150 [130, 540]	162 [142, 209]	153 [133, 185]	0.16
Clear fluid consumption of 200 mL or more, N (%)	3 (6)	4 (6)	5 (3)	0.31
Vomit, N (%)	0 (0)	0 (0)	0 (0)	—
Hypotension, N (%)	3 (6)	1 (2)*	21 (12)*	**0.03**

Bold font: p<0.05, with a significant difference between * and ^#^.

**Table4:** The results for on-call

	Group A (N=39)	Group B (N=43)	Group C (N=102)	P value
Age years	66 [52, 72]*	53 [39, 63]*^#^	63 [52, 74]^#^	**0.002**
Sex Female, N (%)	23 (59)	28 (65)	47 (46)	0.09
Height cm	160 [151, 165]	158 [153, 165]	160 [154, 168]	0.13
Weight kg	54 [48, 63]	56 [50, 60]	57 [51, 69]	0.16
Body Mass Index kg/m^2^	21.8 [20.1, 23.5]	21.6 [20.5, 23.8]	22.7 [20.3, 25.8]	0.78
ASA	2 [1, 2]	1 [1, 2]	2 [1, 2]	0.22
General surgery, N (%)	11 (28)	12 (28)	29 (28)	1
The difference between the scheduled and actual time of entering operation room, min	6 [–30, 36]	15 [2, 54]*	–2 [–51, 16]*	**<0.001**
The difference between the instructed and last drinking time, min	40 [10, 120]	20 [0, 60]	20 [0, 60]	0.09
The duration of fasting, min	296 [242, 359]	291 [252, 363]	274 [219, 360]	0.58
Clear fluid consumption of 200 mL or more, N (%)	5 (13)*	16 (37)*^#^	3 (3)^#^	**<0.001**
Vomit, N (%)	0 (0)	0 (0)	0 (0)	—
Hypotension, N (%)	0 (0)	0 (0)	9 (9)	**0.02**

Bold font: p<0.05, with a significant difference between * and ^#^.

**Table5:** The results of the relationship between the number of days since perioperative management center and the amount of clear fluid consumed

	≤50 mL (N=112)	50–200 mL (N=164)	≥200 mL (N=8)	P value
Number of days since perioperative management center, day	17 [8, 25]	16 [10, 27]	23 [6, 29]	0.81
Preoperative examination Yes, N (%)	44 (39)	76 (46)	5 (63)	0.3

## References

[1] Fearson KC, Ljungqvist O, Von Meyenfeldt M, Revhaug A, Dejong CH, Lassen K, Nygren J, Hausel J, Soop M, Andersen J, Kehlet H. Enhanced recovery after surgery: A consensus review of clinical care for patients undergoing colonic resection. Clin Nutr 2005; 24: 466–477. 15896435 10.1016/j.clnu.2005.02.002

[2] Grant MC, Pio Roda CM, Canner JK, Sommer P, Galante D, Habson D, Gearhart S, Wu CL, Wick E. The impact of anesthesia-influenced process measure compliance on length of stay: Results from an enhanced recovery after surgery for colorectal surgery cohort. Anesth Analg 2019; 128: 68–74. 29782405 10.1213/ANE.0000000000003458

[3] Varadhan KK, Neal KR, Dejong CH, Fearon KC, Ljungqvist O, Lobo DN. The enhanced recovery after surgery (ERAS) pathway for patients undergoing major elective open colorectal surgery: A meta-analysis of randomized controlled trials. Clin Nutr 2010; 29: 434–440. 20116145 10.1016/j.clnu.2010.01.004

[4] Nelson G, Dowdy SC, Lasala J, Mena G, Bakkum-Gamez J, Meyer LA, Iniesta MD, Ramirez PT. Enhanced recovery after surgery (ERAS^®^) in gynecologic oncology—Practical considerations for program development. Gynecol Oncol 2017; 147: 617–620. 28947172 10.1016/j.ygyno.2017.09.023

[5] Batchelor TJP, Rasburn NJ, Abdelnour-Berchtold E, Brunelli A, Cerfolio RJ, Gonzalez M, Ljungqvist O, Petersen RH, Popescu WM, Slinger PD, Naidu B. Guidelines for enhanced recovery after lung surgery: recommendations of the Enhanced Recovery After Surgery (ERAS^®^) Society and the European Society of Thoracic Surgeons (ESTS). Eur J Cardiothorac Surg 2019; 55: 91–115. 30304509 10.1093/ejcts/ezy301

[6] Salenger R, Morton-Bailey V, Grant M, Gregory A, Williams JB, Engelman DT. Cardiac enhanced recovery after surgery: A guide to team building and successful implementation. Semin Thorac Cardiovasc Surg 2020; 32: 187–196. 32120008 10.1053/j.semtcvs.2020.02.029

[7] Brady M, Kinn S, Stuart P. Preoperative fasting for adults to prevent perioperative complications. Cochrane Database Syst Rev 2003: CD004423.10.1002/14651858.CD00442314584013

[8] Carey SK, Conchin S, Bloomfield-Stone S. A qualitative study into the impact of fasting within a large tertiary hospital in Australia—the patients’ perspective. J Clin Nurs 2015; 24: 1946–1954. 25959390 10.1111/jocn.12847

[9] Oshima S, Aoki Y, Kawasaki Y, Yokoyama J. The effect of oral hydration on the risk or aspiration and hemodynamic stability before the induction of anesthesia: A systematic review and meta-analysis. J Clin Anesth 2018; 49: 7–11. 29803011 10.1016/j.jclinane.2018.05.015

[10] Japanese Society of Anesthesiologists. Jutsuzen zetsuinshoku gaidorain (Preoperative fasting guideline); 2012 (in Japanese). <https://anesth.or.jp/files/download/news/20120712.pdf> (Accessed April 29, 2021).

[11] Merchant RN, Chima N, Ljunggvist O, Kok JNJ. Preoperative fasting practices across three Anesthesia societies: Survey of practitioners. JMIR Perioper Med 2020; 3: e15905. 33393934 10.2196/15905 PMC7709845

[12] Dagher C, Tohme J, Chebl RB, Chalhoub V, Richa F, Zeid HA, Madi-Jebara S. Preoperative fasting: Assessment of the practices of Lebanese Anesthesiologists. Saudi J Anaesth 2019; 13: 184–190. 31333361 10.4103/sja.SJA_720_18 PMC6625281

[13] Zhu Q, Li Y, Deng Y, Chen J, Zhao S, Bao K, Lai L. Preoperative Fasting Guidelines: Where Are We Now? Findings From Current Practices in a Tertiary Hospital. J Perianesth Nurs 2021; 36: 388–392. 33678495 10.1016/j.jopan.2020.09.002

[14] Denkyi L. An exploration of pre-operative fasting practices in adult patients having elective surgery. Br J Nurs 2020; 29: 436–441. 32279558 10.12968/bjon.2020.29.7.436

[15] Panebianco A, Laforgia R, Volpi A, Punzo C, Vacca G, Minafra M, Salvo MD, Pezzolla A. Preoperative fasting—“nihil per os” a difficult myth to break down: a randomized controlled study. G Chir 2020; 41: 84–93. 32038017

[16] Kanda Y. Investigation of the freely available easy-to-use software ‘EZR’ for medical statistics. Bone Marrow Transplant 2013; 48: 452–458. 23208313 10.1038/bmt.2012.244 PMC3590441

[17] Erskine L, Hunt JN. The gastric emptying of small volumes given in quick succession. J Physiol 1981; 313: 335–341. 7277223 10.1113/jphysiol.1981.sp013668 PMC1274454

[18] Brener W, Hendrix TR, McHugh P. Regulation of gastric emptying of glucose. Gastroenterology 1983; 85: 76–82. 6852464

[19] Practice guidelines for preoperative fasting and the use of pharmacologic agents to reduce the risk of pulmonary aspiration: application to healthy patients undergoing elective procedures: a report by the American Society of Anesthesiologist Task Force on Preoperative Fasting. Anesthesiology 1999; 90: 896–905. 10.1097/00000542-199903000-00034 10078693

[20] van Noort HHJ, Eskes AM, Vermeulen H, Besselink MG, Moeling M, Ubbink DT, Huisman-de Waal G, Witteman BJM. Fasting habits over a 10-year period: An observational study on adherence to preoperative fasting and postoperative restoration of oral intake in 2 Dutch hospitals. Surgery 2021; 170: 532–540. 33712307 10.1016/j.surg.2021.01.037

[21] Practice guidelines for preoperative fasting and the use of pharmacologic agents to reduce the risk of pulmonary aspiration: Application to healthy patients undergoing elective procedures: an updated report by the American Society of Anesthesiologists Task Force on Preoperative Fasting and the use of pharmacologic agents to reduce the risk of pulmonary aspiration. Anesthesiology 2017; 126: 376–393. 10.1097/ALN.0000000000001452 28045707

[22] Søreide E, Eriksson LI, Hirlekar G, Eriksson H, Henneberg SW, Sandin R, Raeder J. Pre-operative fasting guidelines: an update. Acta Anaesthesiol Scand 2005; 49: 1041–1047. 16095440 10.1111/j.1399-6576.2005.00781.x

[23] Singh BN, Dahiya D, Bagaria D, Saini V, Kaman L, Kaje V, Vagadiya A, Sarin S, Edwards R, Attri V, Jain K. Effects of preoperative carbohydrates drinks on immediate postoperative outcome after day care laparoscopic cholecystectomy. Surg Endosc 2015; 29: 3267–3272. 25609319 10.1007/s00464-015-4071-7

[24] Dock-Nascimento DB, de Aguilar-Nascimento JE, Magalhaes Faria MS, Caporossi C, Slhessarenko N, Waitzberg DL. Evaluation of the effects of a preoperative 2-hour fast with maltodextrine and glutamine on insulin resistance, acute-phase response, nitrogen balance, and serum glutathione after laparoscopic cholecystectomy: a controlled randomized trial. J Parenter Enteral Nutr 2012; 36: 43–52. 10.1177/0148607111422719 22235107

[25] Patil MC, Prajwal B. Randomised clinical trial to compare ultrasonography guided gastric volume in patients after overnight fasting and after ingestion of clear fluids two hours before surgery. Indian Journal of Clinical Anaesthesia 2020; 7: 509–513.

[26] Sessler DI, Bloomstone JA, Aronson S, Berry C, Gan TJ, Kellum JA, Plumb J, Mythen MG, Grocott MPW, Edwards MR, Miller TE. Perioperative quality initiative consensus statement on intraoperative blood pressure, risk and outcomes for elective surgery. Br J Anaesth 2019; 122: 563–574. 30916004 10.1016/j.bja.2019.01.013

[27] Morley AP, Nalla BP, Vamadevan S, Strandvik G, Natarajan A, Prevost AT, Lewis CM. The influence of duration of fluid abstinence on hypotension during propofol induction. Anesth Analg 2010; 111: 1373–1377. 20861421 10.1213/ANE.0b013e3181f62a2b

